# Microsatellites in the Endangered Species *Dyckia distachya* (Bromeliaceae) and Cross-Amplification in Other Bromeliads

**DOI:** 10.3390/ijms131215859

**Published:** 2012-11-27

**Authors:** Camila M. Zanella, Aline Janke, Gecele M. Paggi, Márcia Goetze, Mauricio S. Reis, Fernanda Bered

**Affiliations:** 1 Genetics Department, Biosciences Institute/Federal University of Rio Grande do Sul State – UFRGS, CEP 91501-970, Porto Alegre, RS, Brazil; E-Mails: milamzanella@yahoo.com.br (C.M.Z.); alinejanke@gmail.com (A.J.); marciagoetze@yahoo.com.br (M.G.); 2 Biological Sciences, Pantanal Campus/Federal University of Mato Grosso do Sul State – UFMS, CP 252, CEP 79304-902, Corumbá, MS, Brazil; E-Mail: gecele.paggi@ufms.br; 3 Crop Sciences Department, Agronomy Center, Federal University of Santa Catarina State – UFSC, CP 476, CEP 88040-900, Florianópolis, SC, Brazil; E-Mail: msedrez@gmail.com

**Keywords:** Bromeliad, endemism, introduction, rheophytic species, SSR

## Abstract

Microsatellite markers were isolated in *Dyckia distachya*, an endangered bromeliad from southern Brazil, which will be useful to assess the population genetic structure and reproductive success in introduced and natural populations of this species. Twenty microsatellite loci were developed from an enriched genomic library, and nine of these were amplified. The loci were characterized in 43 individuals from introduced and wild *D. distachya* populations. All nine loci were polymorphic, with four to ten alleles per locus. In an introduced population the observed and expected heterozygosities ranged from 0.136–0.667 and 0.543–0.877, respectively, while in a wild population it ranged from 0.000 to 0.895 and from 0.050 to 0.811, respectively. The development of these microsatellite markers will contribute to investigations of the reproductive potential and viability of introduced populations of *D. distachya* as well as the single known wild population. Cross-amplification in other Bromeliaceae species was successful, with high rates in four loci, demonstrating the applicability of these microsatellite markers in other taxa.

## 1. Introduction

*Dyckia distachya* Hassler is a rare, endemic bromeliad (subfamily Pitcairnioideae) with a rupicolous, rheophytic habit that lives on riverbanks along the rapids of the Pelotas and Uruguay Rivers in southern Brazil [1]. The species is adapted to extreme variations of low and high water periods, and is characterized by high clonal reproduction [2,3]. According to Reitz [1] and Reis *et al*. [2], *D. distachya* is facing extinction due to increased hydroelectric construction along the rivers. Originally, eight wild populations of *D. distachya* were known along the Uruguay and Pelotas Rivers. Currently, only one wild population is known, located in Salto do Yucumã in Turvo State Park, Derrubadas, Rio Grande do Sul State, which is on the border between Brazil and Argentina. Therefore, *D. distachya* is on the Official List of Brazilian Endangered Species [4]. During Barra Grande hydroelectric construction, some *D. distachya* populations were rescued and conserved *ex situ*. However, the process occurred rapidly, without appropriate planning regarding the genetic structure of the populations. Initially, the plants were kept in nurseries before being introduced in environments similar to their origin. In addition to the long-term monitoring of introduced populations, knowledge of the genetic diversity of these populations is needed so that they can be managed appropriately and the success of the introduction is ensured.

Here, we report the isolation and characterization of a set of nine microsatellite loci for *D. distachya* in one introduced population and in the only one known wild population. The cross-amplification of these loci was examined in 22 related species from three subfamilies of Bromeliaceae. The transferability of these microsatellites will be helpful in conservation genetics studies in many bromeliad taxa.

## 2. Results and Discussion

### 2.1. Isolation, Characterization and Cross-Amplification of Microsatellite Loci

Nine from 20 *D. distachya* loci amplified robustly and proved to be polymorphic. Markers isolation involved the development of a genomic enriched library. A total of 96 positive clones were captured and 48 colonies were sequenced, of which 24 clones showed simple sequence repeats (SSR) motifs. Twenty primer pairs were designed in which the product size ranged between 100 and 300 base pairs. Nine of these primer pairs generated consistent patterns of amplification and were used for further characterization of two *D. distachya* populations. The primer sequences and product sizes are shown in Table 1.

In the introduced population, the number of alleles per locus ranged from 4 to 10, with an average of 6.2, and the expected and observed heterozygosities ranged from 0.136 to 0.667 and from 0.543 to 0.877, respectively. In the wild population, the number of alleles per locus varied from 2 to 8, with an average of 3.8, and the expected and observed heterozygosities ranged from 0.000 to 0.895 and from 0.050 to 0.811, respectively (Table 2). Four loci showed significant departures from the Hardy-Weinberg equilibrium (HWE) due to heterozygote deficiency in both populations (Dd04, Dd08, Dd09 and Dd20; Table 2). Such significant deviation from HWE could have been caused by the involvement of null alleles. We tested the presence of null alleles and the four loci that showed significant departures from HWE in both populations exhibited high null allele frequencies (data not shown). Other three loci showed significant departures from HWE in only one population (*p* < 0.001; Table 2) and did not show null alleles, being the departures probably due to inbreeding or Wahlund effects.

Cross-species amplification was successful in different degrees (Table 3). All loci amplified in species from same genus (except Dd19 for *D. maritima*). Locus Dd10 and Dd20 amplified in all species tested, and locus Dd07 and Dd16 were successful in 86.3% of the species evaluated. The 12 genus tested correspond to three representative Bromeliaceae subfamilies from Atlantic Rainforest, indicating high potential for use of these four loci in phylogeography and population genetic studies of many Bromeliaceae species, mainly for *Dyckia* genus.

### 2.2. Genetic Diversity of D. distachya

In our study, introduced population showed, on average, a higher number of alleles per locus, and higher observed and expected heterozygosities compared with the wild population (Table 2). The results could indicate that the individuals rescued may be representative of the original population, retaining the genetic diversity, although further studies should be made for a more accurate conclusion. The introduced population presented 25 exclusive alleles of a total 60 alleles (data not show). The wild population showed only three exclusive alleles and low genetic diversity. The microsatellites characterized here will be used to assess more thoroughly the genetic diversity of natural and introduced populations. However, one hypothesis for lower genetic diversity found in natural population would be the edge effect [5], since this population occurs at the range edge of the original geographical distribution of the species. This phenomenon has also been observed in *Vriesea gigantea*, another species of bromeliad [6].

## 3. Experimental Section

### 3.1. Isolation of Microsatellite Markers

To identify and characterize microsatellites, genomic DNA was extracted from fresh leaves of one *D. distachya* individual following the protocol of Doyle and Doyle [7]. Microsatellite markers were developed as described by Kijas *et al*. [8] with modifications by Billote *et al*. [9], using an enriched library methodology. In brief, total DNA was cleaved with the restriction enzyme *Rsa*I (Invitrogen, Carlsbad, CA, USA). The cleaved DNA was linked to two complementary oligo adapters. Polymerase chain reaction (PCR) was performed using one of the adapters as a primer. The PCR product was purified using a *QIAquick PCR* Purification Kit (Qiagen, Hilden, Germany). Fragments that contained microsatellite repeats were selected using biotinylated probes (dCT)_8_ and (dGT)_8_ and isolated with *Dynabeads M-280 Streptavidin* magnetic particles (Invitrogen, Carlsbad, CA, USA). An additional PCR was performed using one of the adapters as a primer. The PCR products were inserted into the *pGEM-T Easy* vector (Promega, Madison, WI, USA) and used to transform XL1-Blue competent cells (*Escherichia coli*, Stratagene, La Jolla, CA, USA). Recombinant colonies were selected by blue/white screening. Ninety-six recombinant colonies were randomly selected, and 48 were double-sequenced using the *BigDye* Terminator version 3.1 Cycle Sequencing Kit (Applied Biosystems, Foster City, CA, USA) on an *ABI PRISM 377* DNA Sequencer (Applied Biosystems, Foster City, CA, USA). Every the forward and reverse sequences obtained were aligned and edited in *SeqMan* (DNASTAR package, Madison, WI, USA), and primers were designed using the *Primer3* program [10].

### 3.2. Primer Validation

To assess polymorphisms, 22 individuals from one *D. distachya* population introduced in Anita Garibaldi, Santa Catarina State, Brazil (27°58′81″S, 50°39′11″W) and 21 individuals from the wild population were genotyped. The wild population was collected in Salto do Yucumã, in Turvo State Park, Derrubadas, Rio Grande do Sul State (27°09′62″S, 53°53′45″W), southern edge of species original distribution and the introduced population came from the *ex situ* collection.

For each SSR, the forward primers were synthesized with a 19-bp M13 tail (5′-CACGACGTTGTAAAACGAC-3′) following the method of Schuelke [11], which involved three primers: a forward SSR-specific primer with the M13 tail at its 5′ end, a reverse locus-specific primer, and a universal M13 primer labeled with one of four fluorescent dyes (6-FAM, VIC, NED, and PET; Applied Biosystems, Foster City, CA, USA). All PCR amplifications were performed in a *TC-412* thermal cycler (Techne, Burlington, NJ, USA) in 10 μL reactions containing 10 ng of DNA template, 1× *Taq* buffer, 2 mM MgCl_2_, 200 μM of each dNTP, 1 pmol forward primer, 4 pmol reverse primer, 0.4 pmol universal M13 primer, and 0.5 U *Taq* DNA polymerase (*GoTaq*; Promega, Madison, WI, USA). The touchdown cycling program was used: 95 °C for 3 min, 10 cycles of 94 °C for 30 s, 58 °C decreasing to 48 °C at 1 °C per cycle for 30 s, 72 °C for 30 s, followed by 30 cycles of 94 °C for 30 s, 48 °C for 30 s, and 72 °C for 30 s, and concluding with a 10 min extension at 72 °C. The microsatellite alleles were resolved on a *ABI 3100* DNA Analyzer Sequencer (Applied Biosystems, Foster City, CA, USA) and sized against the *GS500 LIZ* molecular size standard (Applied Biosystems, Foster City, CA, USA) using GeneMarker DEMO version 1.97 (SoftGenetics, State College, PA, USA). The number of alleles, and expected and observed heterozygosities were analyzed with MSA 4.00 software [12], and Genepop 4.1 [13] was used to test for departure from the Hardy-Weinberg equilibrium (HWE). Null allele frequencies were calculated following [14] for each population, using the software Micro-Checker version 2.2.3 [15].

The polymorphic markers were also tested for cross-transferability in 22 related species from three subfamilies of Bromeliaceae. The PCR conditions were the same as described above. The amplification products were separated on 2% agarose gels (Agarose LE; USB, Cleveland, OH, USA) stained with *GelRed* (Biotium, Hayward, CA, USA) and visualized under ultraviolet light. A locus was considered to have been amplified successfully when at least one band of the expected size was visualized. A 50-bp *DNA* ladder (Ludwig Biotec, Alvorada, RS, Brazil) was used as a molecular size marker. All voucher specimens of the species investigated are identified in Table S1.

## 4. Conclusions

In the present study, microsatellite-enriched genomic library of *Dychia distachya* was constructed and a total of nine polymorphic microsatellite DNA markers were characterized. The development of these markers will contribute to future population genetic studies in *D. distachya* and other bromeliad species. The nine loci will also be useful for identifying the effects of introduction on outcrossing and selfing rates, with a comparative analysis among introduced and wild populations of *D. distachya* using progeny arrays. The use of such markers will facilitate inferences about the reproductive potential and viability of the introduced *D. distachya* populations and will aid in the understanding of the role of genetics in plant species introduction.

## Supplementary Information



## Figures and Tables

**Table 1:** Description and characterization of nine microsatellite loci of *Dyckia distachya*, including locus name, primer sequences, repeat motif, number of alleles (A), allele size range and GeneBank accession number.

Locus	Primer Sequences (5′-3′)	Repeat motif	A	Size (bp)	GeneBank accession No
Dd03	F: TAGGCAGATGCGAATTGAGT R: CTCAGCATAGCTTTCGATGG	(CA)_10_	6	202–212	JX290116
Dd04	F: CGCTTTTCTCGGAATCTTTG R: AGGGCTTCGTCCTCCTAAAA	(TG)_8_	6	227–257	JX290117
Dd07	F: GATTCGGAAGGATGACAAGA R: CGGCACAGGAATACAAAGAG	(GA)_25_	5	201–213	JX290118
Dd08	F: GATCGGTCTTTTACTCCTTGG R: CACGCTAGGATGATGTAGGC	(AC)_6_G(GA)_9_	10	192–232	JX845415
Dd09	F: ACTCTGCTGCCTCATTCACA R: CACAGCAAAGGCAAACTTGA	(GA)_10_	10	176–228	JX845416
Dd10	F: CTATGGGACTGCTGGACACT R: CTTGCTGGTAATCGTGTTCC	(TG)_7_	4	248–254	JX290119
Dd16	F: AATTGCACCAAACAGGGATT R: GACACGACCCACATAGATGC	(GT)_7_ (GC)_4_	7	170–190	JX290120
Dd19	F: GTGCCAAACATAAACCGATG R: AACGACCAAAAAGGGTGTTC	(GT)_5_	6	239–278	JX290121
Dd20	F: GGTGGAAATGGTGGGTTACA R: GGCAGGCAAGGTATGAAGAA	(CA)_7_	6	227–253	JX290122

**Table 2:** Results of nine microsatellite loci in two populations of *Dyckia distachya*, including number of individuals (*N*), number of alleles (*A*), observed (*H*_O_) and expected (*H*_E_) heterozygosities.

Locus	Introduced (*N* = 22)			Natural (*N* = 21)		
	
	*A*	*H*_O_	*H*_E_	*A*	*H*_O_	*H*_E_
Dd03	6	0.636	0.724*	3	0.350	0.309
Dd04	6	0.136	0.636*	3	0.214	0.659*
Dd07	4	0.667	0.582	2	0.050	0.050
Dd08	10	0.300	0.877*	7	0.300	0.720*
Dd09	8	0.136	0.558*	8	0.167	0.811*
Dd10	4	0.545	0.589	3	0.143	0.257
Dd16	6	0.545	0.601	4	0.895	0.552*
Dd19	6	0.182	0.543*	2	0.048	0.136
Dd20	6	0.476	0.784*	3	0.000	0.284*
Mean	6.2	0.402	0.591	3.8	0.241	0.188

* Indicates H_O_ departed significantly from *H*_E_ under HWE (*p* < 0.001).

**Table 3:** Cross-amplification of nine microsatellite markers isolated from *Dyckia distachya* across three subfamilies of Bromeliaceae.

Species	Subfamily	Dd03	Dd04	Dd07	Dd08	Dd09	Dd10	Dd16	Dd19	Dd20
*Dyckia leptostachya*	Pitcairnioideae	+	+	+	+	+	+	+	+	+
*Dyckia maritima*	Pitcairnioideae	+	+	+	+	+	+	+	−	+
*Dyckia tuberosa*	Pitcairnioideae	+	+	+	+	+	+	+	+	+
*Acanthostachys strobilacea*	Bromelioideae	−	−	+	−	−	+	+	−	+
*Aechmea caudata*	Bromelioideae	−	−	+	−	−	+	+	−	+
*Aechmea coelestis*	Bromelioideae	−	−	−	−	−	+	+	−	+
*Aechmea comata*	Bromelioideae	w	−	+	−	−	+	+	−	+
*Aechmea gamosepala*	Bromelioideae	−	−	+	+	−	+	+	−	+
*Aechmea recurvata*	Bromelioideae	+	−	+	−	−	+	+	−	+
*Aechmea winkleri*	Bromelioideae	w	−	+	w	−	+	+	−	+
*Billbergia amoena*	Bromelioideae	−	−	w	−	+	+	+	−	+
*Bromelia antiacantha*	Bromelioideae	−	−	+	−	+	+	+	+	+
*Edmundoa lindenii*	Bromelioideae	−	−	−	−	−	+	+	−	+
*Hohenbergia augusta*	Bromelioideae	−	−	−	w	+	+	+	−	+
*Neoregelia guttata*	Bromelioideae	+	−	+	+	+	+	+	−	+
*Nidularium procerum*	Bromelioideae	+	−	+	+	−	+	+	w	+
*Quesnelia quesneliana*	Bromelioideae	−	−	+	w	−	+	+	−	+
*Alcantarea extensa*	Tillandsioideae	+	−	+	w	−	+	+	−	+
*Vriesea carinata*	Tillandsioideae	+	−	+	−	−	+	w	+	+
*Vriesea gigantea*	Tillandsioideae	+	−	+	−	−	+	−	−	+
*Vriesea incurvata*	Tillandsioideae	+	−	+	−	−	+	−	−	+
*Vriesea reitzii*	Tillandsioideae	+	−	+	−	−	+	−	−	+
Total = 22		13	3	19	10	7	22	19	5	22

Note: + = successful PCR amplification; W = weak PCR amplification; − = PCR failure.

## References

[1] Reitz R (1983). Bromeliáceas e a malária—Bromélia endêmica. Flora Ilustrada Catarinense.

[2] Reis A., Rogalski J., Vieira N.K., Berkenbrock I.S. (2005). Conservação de espécies reófitas de *Dyckia* do sul do Brasil: *Dyckia distachya*; Technical Report.

[3] Wiesbauer M.B., Reis A, Tres D.R., Reis A. (2009). Conservação *ex situ* e reintrodução de espécies na natureza: O que aprendemos nas experiências com a reófita *Dyckia distachya*. Perspectivas sistêmicas para a conservação e restauração ambiental: do pontual ao contexto.

[4] MMA—Ministério do Meio Ambiente (Ministry of Environment) (2008). Normative Statement No. 6.

[5] Eckert C.G., Samis K.E., Lougheed S.C. (2008). Genetic variation across species’ geographical ranges: The central–marginal hypothesis and beyond. Mol. Ecol.

[6] Palma-Silva C., Lexer C., Paggi G.M., Barbará T., Bered F., Bodanese-Zanettini M.H. (2009). Range-wide patterns of nuclear and chloroplast DNA diversity in *Vriesea gigantea* (Bromeliaceae), a neotropical forest species. Heredity.

[7] Doyle J.J., Doyle J.L. (1990). Isolation of plant DNA from fresh tissue. Focus.

[8] Kijas J.M., Fowler J.C., Garbett C.A., Thomas M.R. (1994). Enrichment of microsatellites from the citrus genome using biotinylated oligonucleotide sequences bound to streptavidin-coated magnetic particles. BioTechniques.

[9] Billote N., Risterucci A.M., Baurens F.C. (1999). Microsatellite enriched libraries: Applied methodology for the development of SSR markers in tropical crops. Fruits.

[10] Rozen S., Skaletsky H, Krawetz S., Misener S. (2000). Primer3 on the WWW for General Users and for Biologist Programmers. *Bioinformatics Methods and Protocols* in the series *Methods in Molecular Biology*.

[11] Schuelke M. (2000). An economic method for the fluorescent labeling of PCR fragments. Nat. Biotechnol.

[12] Dieringer D., Schlötterer C. (2003). Microsatellite Analyser (MSA): A platform independent analysis tool for large microsatellite data sets. Mol. Ecol. Notes.

[13] Raymond M., Rosset F. (1995). Genepop: Population genetics software for exact test and ecumenicism. J. Hered.

[14] Brookfield J.F.Y. (1996). A simple new method for estimating null allele frequency from heterozygote deficiency. Mol. Ecol.

[15] Van Oosterhout C., Hutchinson W.F., Wills D.P.M., Shipley P. (2004). MICRO-CHECKER: Software for identifying and correcting genotyping errors in microsatellite data. Mol. Ecol. Notes.

