# Supplementary material for: Microsatellites in the Endangered Species Dyckia distachya (Bromeliaceae) and Cross-Amplification in Other Bromeliads
Source: Int J Mol Sci. 2012 Nov 27;13(12):15859–66. doi: 10.3390/ijms131215859 (PMC3546666; doi:10.3390/ijms131215859)
Supplement: Supplementary file 1 [file ijms-13-15859-s001.pdf]

Supplementary Information

Table S1. Information on voucher specimens deposited in MBLM (Melo Leitão Herbarium), FLOR (Botanic Department Herbarium of Universidade Federal de Santa Catarina), SP (Maria Eneyda P. K. Fidalgo Herbarium), ICN (Universidade Federal do Rio Grande do Sul Herbarium), HBR (Barbosa Rodrigues Herbarium), HAS (Alarich Rudolf Holger Schultz Herbarium), HCOR (Universidade Federal de Mato Grosso do Sul Herbarium). Species: herbarium acronyms and voucher number. BRA – Brazil.

Acanthostachys strobilacea (Schult. f.) Klotzsch.: MBML 10227, BRA, Espírito Santo, Santa Teresa.	*Dyckia marítima* Baker: ICN 127214, BRA, Rio Grande do Sul, Viamão.
Aechmea caudata Lindm.: FLOR 39079, BRA, Santa Catarina, Florianópolis.	*Dyckia tuberosa* (Vellozo) Beer: HAS 30828, BRA, Rio Grande do Sul, Quaraí.
Aechmea coelestis (K. Kock) E Morren: SP 374431, BRA, São Paulo, Ubatuba.	*Edmundoa lindenii* (Regel) Leme: MBML 14205, BRA, Espírito Santo, Santa Teresa.
Aechmea comata Baker: ICN 165256, BRA, Santa Catarina, Florianópolis.	*Hohenbergia augusta* (Vell.) E. Morren: MBML 14390, BRA, Espírito Santo, Santa Teresa.
Aechmea gamosepala Wittm.: ICN 165259, BRA, Rio Grande do Sul, Torres.	*Neoregelia guttata* Leme. MBML 12622, BRA, Espírito Santo, Santa Teresa.
Aechmea recurvata (Klotzsch) L.B. Sm.: ICN 165344, BRA, Rio Grande do Sul, Viamão.	*Nidularium procerum* Lindm.: MBML 14917, BRA, Espírito Santo, Santa Teresa.
Aechmea winkleri Reitz: ICN 189267, BRA, Rio Grande do Sul, Santa Cruz do Sul.	*Quesnelia quesneliana* (Brongn.) L.B. Sm.: MBML 24974, BRA, Espírito Santo, Santa Teresa.
Alcantarea extensa (L.B. Sm.) J.R. Grant.: MBML 25417, BRA, Espírito Santo, Santa Teresa.	*Vriesea carinata* Wawra: MBML 14361, BRA, Espírito Santo, Santa Teresa.
Bilbergia amoena (Lodd.) Lindley: MBML 24913, BRA, Espírito Santo, Santa Teresa.	*Vriesea gigantea* Gaudich.: ICN 115410, BRA, Rio Grande do Sul, Viamão.
Bromelia antiacantha Bertoloni: HBR 4067, BRA, Santa Catarina, Mafra.	*Vriesea incurvata* Gaudich.: HBR 4097, BRA, Santa Catarina, Florianópolis.
Dyckia distachya Hassl.: HAS 37763, BRA, Rio Grande do Sul, Machadinho.	*Vriesea reitzii* Leme & Costa, Andrea: HAS 66298, BRA, Rio Grande do Sul, São Francisco de Paula.
Dyckia leptostachya Baker: HCOR 13576, BRA, Mato Grosso do Sul, Ladário.	

© 2012 by the authors; licensee MDPI, Basel, Switzerland. This article is an open access article distributed under the terms and conditions of the Creative Commons Attribution license (http://creativecommons.org/licenses/by/3.0/).
